# Incidence, Mortality, and Survival Trends of Liver Cancer in the Canton of Zurich, Switzerland, Between 1980 and 2022

**DOI:** 10.1002/cam4.72393

**Published:** 2026-10-21

**Authors:** Nina Zuber, Flurina Suter, Miriam Wanner, Dimitri Korol, Sabine Rohrmann

**Affiliations:** ^1^ Cancer Registry of the Canton Zurich, Zug, Schaffhausen and Schwyz University Hospital Zurich Zurich Switzerland; ^2^ Division of Chronic Disease Epidemiology, Epidemiology, Biostatistics, and Prevention Institute University of Zurich Zurich Switzerland

## Abstract

**Background:**

The global burden of liver cancer has been increasing over recent decades, whereas the incidence in Switzerland has remained relatively stable. This study assessed long‐term trends in incidence, mortality, and 1‐ and 5‐year relative survival of primary liver cancer in the Swiss canton of Zurich from 1980 to 2022, stratified by sex and age.

**Methods:**

This population‐based retrospective analysis included all primary liver cancer cases diagnosed in the canton of Zurich and registered in the Cancer Registry of the cantons of Zurich, Zug, Schaffhausen and Schwyz between 1980 and 2022. Mortality data were obtained from the Federal Statistical Office. Cases were classified according to ICD‐O‐3 topography and morphology and, from 2003 onward, TNM stage. Age‐standardised incidence and mortality rates were calculated using the 1976 European standard population. Temporal trends were analysed using Joinpoint regression and reported as Average Annual Percentage Changes (AAPCs) with 95% confidence intervals (CIs). Relative survival was estimated using the Ederer II method and standardised according to international standards.

**Results:**

Among 3866 incident cases, 71.3% were hepatocellular carcinomas and 70.7% occurred in men. Overall age‐standardised incidence remained stable in both men (AAPC −0.1%, 95% CI −0.6 to 0.3%) and women (−0.2%, −0.9% to 0.4%). However, incidence increased significantly among individuals younger than 65 years, particularly in women (AAPC 2.3%, 1.3%–3.3%) and men (1.2%, 0.6%–1.9%). Mortality remained broadly stable in men and increased modestly in women. One‐ and five‐year relative survival improved substantially, from 13.8% and 3.5% in 1980–1999 to 53.9% and 25.8% in 2016–2022, respectively. Improvements were greater in men than women.

**Conclusion:**

These findings reflect major advances in liver cancer diagnosis and treatment over the past four decades. Continued prevention, targeted screening of high‐risk groups, and epidemiological monitoring remain important.

## 1 Introduction

Liver cancer continues to pose a significant threat to global health, with projections indicating over one million new cases per year by 2040 [1]. Hepatocellular carcinoma (HCC) represents the predominant form of this malignancy, comprising approximately 90% of all liver cancer diagnoses [2]. Established risk factors for HCC include chronic hepatitis B and C infections, alcohol consumption, steatotic liver disease (SLD), and exposure to certain toxins such as aflatoxin [3]. The relative contribution of these risk factors varies substantially according to geographical region and population characteristics [4]. In recent years, the nomenclature of fatty liver disease has been revised. According to the 2023 multisociety Delphi consensus, SLD is the overarching term, while metabolic dysfunction‐associated steatotic liver disease (MASLD) replaces the former term non‐alcoholic fatty liver disease (NAFLD), and metabolic dysfunction‐associated steatohepatitis (MASH) replaces non‐alcoholic steatohepatitis (NASH) [5]. MASLD is defined by the presence of hepatic steatosis together with at least one cardiometabolic risk factor. Epidemiologically, contrasting trends are observed: While HCC incidence is declining in some Asian countries due to successful hepatitis B vaccination programmes, it is rising in many parts of the world, including the United States, Australia, and most parts of Europe, driven by the increasing prevalence of non‐alcoholic fatty liver disease [1, 6].

Liver cancer generally has low survival [7]. One reason for the poor prognosis is that it is often diagnosed at a more advanced stage. In early stages of the disease, surgical removal is the standard treatment. Only 5%–15% of all liver cancer patients are diagnosed at an early stage and therefore qualify for surgical resection. For more advanced stages, treatment options include trans‐arterial chemoembolization and chemotherapeutics [8].

According to the Global Cancer Statistics 2022, liver cancer ranks as the sixth most commonly diagnosed cancer worldwide, accounting for 4.3% of all new cancer cases, and the third leading cause of cancer‐related deaths, responsible for 7.8% of all such deaths [9]. In Switzerland, liver cancer accounted on average for 11.6% (men) and 3.6% (women) of all newly diagnosed cancer cases in Switzerland in the years 2017–2021. Meanwhile, liver cancer accounted for 7.8% of all cancer‐related deaths among men and 2.8% among women [10].

The aim of this study was to analyse incidence, mortality, one‐year and five‐year relative survival of liver cancer in the Swiss canton of Zurich between 1980 and 2022.

## 2 Methods

The study followed the outline of the Reporting of studies Conducted using Observational Routinely collected Data (RECORD) guidelines [11].

### 2.1 Data Sources, Inclusion Criteria and Study Design

We performed a retrospective analysis using data for the canton of Zurich from the Cancer Registry of the cantons of Zurich, Zug, Schaffhausen, and Schwyz (CRZZSS) and the Federal Statistical Office (FSO). The CRZZSS contains data on liver cancer patients residing in the canton of Zurich from 1980 to 2022. Death statistics for liver cancer are available at the FSO.

Since 1980, the cantonal cancer registry has systematically collected and documented data on all cancer cases diagnosed among residents of the canton of Zurich at the time of diagnosis. The registry ensures a comprehensive recording of newly diagnosed cancer cases within the population for epidemiological monitoring and cancer research purposes. Reporting of such cases is mandatory by law since 2020 and involves the submission of detailed clinical and pathological information by healthcare providers, while adhering to strict confidentiality and data protection regulations. Before 2020, the registration was based on a decision of the Zurich Government Council from 1980 and the Cantonal Cancer Registration Law from 2017. The registry's work supports the evaluation of cancer incidence, treatment outcomes, and the effectiveness of preventive measures within the canton. This organised collection and analysis of data is aligned with national standards under the Swiss Cancer Registration Act to ensure consistency and quality in cancer surveillance [12, 13, 14].

The incidence analysis was based on primary liver cancer cases (C22.0 and C22.1 according to the International Classification of Diseases for Oncology, Third Edition (ICD‐O‐3) [15]) diagnosed in the canton of Zurich between 1980 and 2022. Cases were excluded if the date of birth was missing, if a death certificate was the only source of information, or if they were lost to follow‐up.

Liver cancer cases were categorised into five histological subgroups based on the ICD‐O‐3 [15] morphology codes: HCC (8170, 8171, 8172, 8174); intrahepatic cholangiocarcinoma (iCCA) (8140, 8160, 8161, 8560, 8576); combined hepatocellular and intrahepatic cholangiocarcinoma (8180); other specified histology (8010, 8020, 8033, 8041, 8162, 8240, 8246, 8840, 8890, 8910, 8970, 8991, 9120, 9130, 9133); and unspecified histology (8000).

Liver cancer cases were staged according to the TNM classification of malignant tumours (TNM) (I, II, III, IV or ‘missing’). Staging analysis included cases diagnosed from 2003 to 2022, as systematic tumour stage documentation was not available prior to 2003. TNM editions were assigned by year of diagnosis: sixth edition (TNM6) for 2003–2009; seventh edition (TNM7) for 2010–2017; and eighth edition (TNM8) for 2018–2022. Where pathological metastasis (pM) data were unavailable, clinical data (cM) were used, and vice versa; this approach was applied consistently to node and tumour data (pN, cN, pT, cT). When both clinical and pathological data were available for node, metastasis, or tumour, but discordant, pathological data were prioritised for analysis. In cases where neoadjuvant therapy was administered, clinical TNM was used.

Histological subgroups and staging were used only for incident case characterisation. Incidence and survival trends for these subgroups were not analysed because the number of cases within each subgroup was limited. The data included information regarding sex of the patient (male, female), year of diagnosis (1980–1999, 2000–2015, 2016–2022), age at diagnosis (< 65 years, 65–74 years, ≥ 75 years), topography (C22.0, C22.1) and morphology (HCC, iCCA, cHCCiCCA, unspecified, other specified) according to ICD‐O‐3, basis of diagnosis, vital status at last follow‐up (alive, dead, lost to follow‐up), year of last follow‐up, age at last follow‐up, and administration of neoadjuvant therapy (yes, no).

The mortality data used from the FSO included all registered deaths in the canton of Zurich for the years 1980–2022. The dataset contained the year of birth, year of death, sex, and the main cause of death. The main cause of death was coded according to the International Classification of Diseases, 8th revision (ICD‐8) [16] up to 1994 and according to the 10th revision (ICD‐10) from 1995 onward. For the mortality analysis, all cases with ICD codes C22.0, C22.1 and 155 were included.

### 2.2 Statistical Analysis

Characteristics of incident liver cancer cases and liver cancer‐related deaths were analysed descriptively. Categorical variables were displayed as absolute numbers and percentages, overall and stratified by sex.

Age‐standardised incidence and mortality rates per 100,000 person‐years were calculated using the 1976 European standard population [17] and population estimates from the canton of Zurich. Due to the unavailability of population data for 1980, population data from 1981 were used instead. Incidence and mortality rates for liver cancer were determined overall and stratified by sex, age group, and period of diagnosis or death.

Statistical analyses were conducted using R version 4.5.2 [18]. Temporal trends in incidence and mortality were assessed with Joinpoint Trend Analysis Software (Version 5.4.0.0 [19]). The Joinpoint model initially assumed zero joinpoints (indicating a single linear trend), and additional joinpoints were tested sequentially and retained only if they significantly improved the model fit, assessed by permutation tests with Bonferroni‐adjusted *p*‐values to compare model fits [20]. Following the Joinpoint software manual recommendations [21], the maximum number of joinpoints was limited to five. A minimum of two observations was required between each joinpoint and at the dataset's boundaries, including when a joinpoint coincided with an observation.

The dependent variables were age‐standardised incidence and mortality rates, with calendar year as the independent variable. The models estimated annual percentage changes (APC) for each linear trend segment and the average annual percentage change (AAPC) as a summary measure for the entire observation period (1980–2022). Because the data were not normally distributed, dependent variables were log‐transformed. AAPC estimates are reported with corresponding 95% confidence intervals (CI) [22].

Survival time was defined as age at death or last‐follow‐up, whichever date occurred first, minus the age at diagnosis. Age‐standardised one‐year and five‐year relative survival were estimated as the ratio of observed to expected survival, irrespective of the cause of death. Expected survival was derived using the Ederer II method, based on lifetables. Relative survival estimates were age‐standardised according to the International Cancer Survival Standards [23], applying standard weights for the following age groups: 0.07 (0–44 years), 0.12 (45–54 years), 0.23 (55–64 years), 0.29 (65–74 years), and 0.29 (≥ 75 years).

## 3 Results

Between 1980 and 2022, a total of 4091 incident liver cancer cases were recorded in the canton of Zurich, Switzerland. Of these, two cases were excluded due to missing birth date information, 210 cases were excluded because a death certificate was the only source of information, and 13 cases were lost to follow‐up. In total, 3866 incident liver cancer cases met the inclusion criteria for the present study. During the same period, 3381 liver cancer deaths were registered. Tables 1 and 2 describe the cases by sex, diagnostic period, age at diagnosis, topography code, histological subgroup, and cancer stage for incidence and by sex, period of death, and age at death for mortality.

**TABLE 1:** Absolute number and percentage of liver cancer cases, overall and by sex, from 1980 to 2022 in the canton of Zurich, Switzerland.

Variables	Overall, *n* (%)	Men, *n* (%)	Women, *n* (%)
*n*	3866	2735	1131
Age at diagnosis (years)			
< 65	1324 (34.2)	981 (35.9)	343 (30.3)
65–74	1222 (31.6)	908 (33.2)	314 (27.8)
≥ 75	1320 (34.1)	846 (30.9)	474 (41.9)
Year of diagnosis			
1980–1999	1360 (35.2)	984 (36.0)	376 (33.2)
2000–2015	1615 (41.8)	1137 (41.6)	478 (42.3)
2016–2022	891 (23.0)	614 (22.4)	277 (24.5)
Topography			
C220	3113 (80.5)	2360 (86.3)	753 (66.6)
C221	753 (19.5)	375 (13.7)	378 (33.4)
Morphology			
HCC^a^	2757 (71.3)	2132 (78.0)	625 (55.3)
iCCA^b^	748 (19.3)	379 (13.9)	369 (32.6)
cHCCiCCA^c^	63 (1.6)	41 (1.5)	22 (1.9)
Unspecified	212 (5.5)	137 (5.0)	75 (6.6)
Other specified	86 (2.2)	46 (1.7)	40 (3.5)
Stage^d^			
I	269 (11.9)	196 (12.6)	73 (10.5)
II	219 (9.7)	167 (10.7)	52 (7.5)
III	127 (5.6)	89 (5.7)	38 (5.5)
IV	315 (14.0)	201 (12.9)	114 (16.4)
Missing	1327 (58.8)	907 (58.1)	420 (60.3)

^a^ Hepatocellular carcinoma.

^b^ Intrahepatic cholangiocarcinoma.

^c^ Combined hepatocellular and intrahepatic cholangiocarcinoma.

^d^ Cancer‐stage analysis was performed for the period between 2003 and 2022 because the stages were not registered systematically prior to 2003.

**TABLE 2:** Absolute number and percentage of deaths due to liver cancer, overall and by sex, from 1980 to 2022 in the canton of Zurich, Switzerland.

Variables	Overall, *n* (%)	Men, *n* (%)	Women, *n* (%)
*n*	3381	2222	1159
Age at death (years)			
< 65	876 (25.9)	630 (28.4)	246 (21.2)
65–74	1000 (29.6)	703 (31.6)	297 (25.6)
≥ 75	1505 (44.5)	889 (40.0)	616 (53.1)
Year of death			
1980–1999	1159 (34.3)	794 (35.7)	365 (31.5)
2000–2015	1479 (43.7)	955 (43.0)	524 (45.2)
2016–2022	743 (22.0)	473 (21.3)	270 (23.3)

### 3.1 Incidence

Between 1980 and 1999, the overall age‐standardised incidence of liver cancer was 7.4 per 100,000 person‐years in men and 2.8 in women. In the most recent period (2016–2022), the corresponding rates were 8.6 in men and 3.7 in women. Over the entire observation period from 1980 to 2022, the AAPC was −0.1% (95% CI −0.6% to 0.3%) among men and −0.2% (95% CI −0.9% to 0.4%) among women, indicating that the overall incidence of liver cancer remained largely stable over time. Age‐standardised incidence rates were consistently higher in men than in women across all periods (Table 3, Figure 1).

**TABLE 3:** Age‐standardised incidence and mortality rates of liver cancer (per 100,000 person‐years), by sex and period, and age‐standardised one‐year and five‐year relative survival of liver cancer, by sex and period, from 1980 to 2022 in the canton of Zurich, Switzerland.

Variables	ASIR^a^		
Period	Overall	Men	Women
1980–1999	5.1	7.4	2.8
2000–2015	6.3	9.0	3.5
2016–2022	6.1	8.6	3.7

Variables	ASMR^b^		
Period	Overall	Men	Women
1980–1999	4.4	6.8	2.0
2000–2015	4.8	6.9	2.7
2016–2022	4.3	6.0	2.7

Variables	AS1YR^c^		
Period	Overall, % [95% CI^d^]	Men, % [95% CI^d^]	Women, % [95% CI^d^]
1980–1999	13.8 [12.0, 15.9]	13.0 [10.9, 15.4]	16.5 [13.1, 20.8]
2000–2015	40.0 [37.5, 42.5]	39.8 [37.0, 43.0]	40.1 [35.7, 45.0]
2016–2022	53.9 [50.6, 57.4]	56.0 [51.9, 60.3]	49.9 [44.4, 56.1]

Variables	AS5YR^e^		
Period	Overall, % [95% CI^d^]	Men, % [95% CI^d^]	Women, % [95% CI^d^]
1980–1999	3.5 [2.5, 4.8]	2.5 [1.6, 3.9]	6.0 [3.9, 9.2]
2000–2015	16.2 [14.3, 18.5]	16.5 [14.2, 19.3]	15.2 [12.0, 19.4]
2016–2022	25.8 [22.5, 29.6]	27.9 [23.8, 32.8]	21.9 [17.0, 28.2]

^a^ Age‐standardised incidence rate.

^b^ Age‐standardised mortality rate.

^c^ Age‐standardised one‐year relative survival.

^d^ Confidence interval.

^e^ Age‐standardised five‐year relative survival.

**FIGURE 1:**
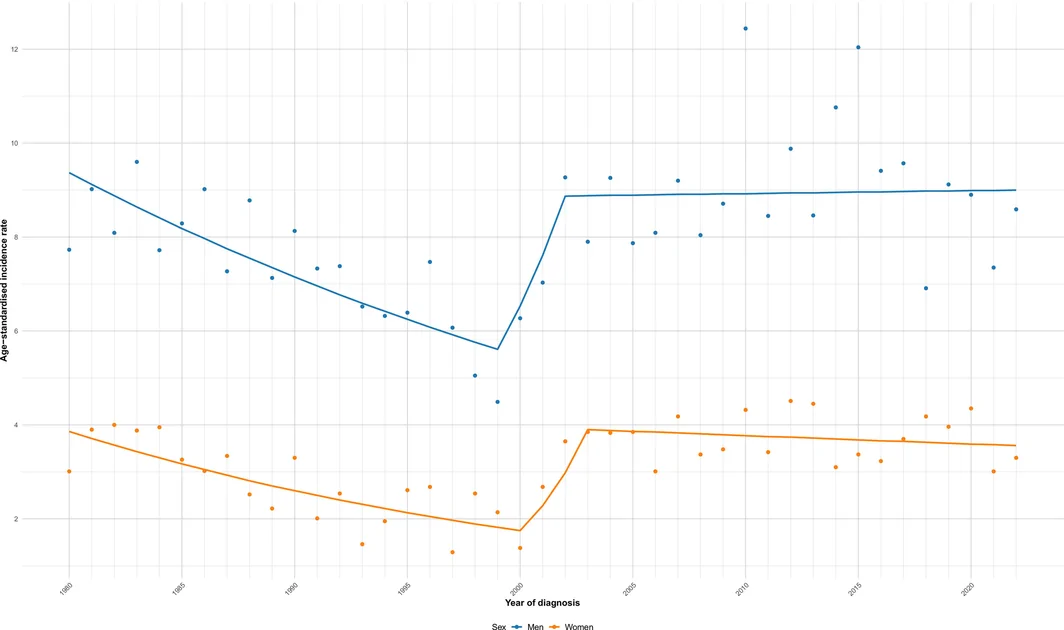
Trends in age‐standardised incidence rates for liver cancer (per 100,000 person‐years) from 1980 to 2022, by sex, in the canton of Zurich, Switzerland. The points show the observed values, and the linear trend line indicates the average percentage change.

Joinpoint regression analysis identified two significant joinpoints for both sexes. In men, a significant decline in incidence rates was observed up to 1999, followed by a significant increase until 2002, and a subsequent stable trend. Among women, the trend showed a significant decrease until 2000, a significant increase until 2003, and a non‐significant decline in the following years (Table 4, Figure 1). Despite these temporal fluctuations, long‐term trends suggest relatively stable age‐standardised incidence rates over the four‐decade study period.

**TABLE 4:** Annual percentage changes for incidence and mortality by sex and age.

Variables	Men	Women
**Incidence**		
All ages	Period: APC^a^ [95% CI^b^]	Period: APC^a^ [95% CI^b^]
	1980–1999: −2.65 [−4.29, −1.60]	1980–2000: −3.89 [−6.15, −2.41]
	1999–2002: 16.45 [2.62, 22.56]	2000–2003: 30.72 [6.52, 41.15]
	2002–2022: 0.07 [−2.01, 1.01]	2003–2022: −0.48 [−2.88, 0.97]
Age < 65 years	1980–2022: 1.23 [0.55, 1.93]	1980–2022: 2.33 [1.32, 3.34]
Age 65–74 years	1980–2022: 0.75 [−0.09, 1.59]	1980–1982: 70.74 [−3.09, 178.99]
		1982–1998: −5.99 [−32.24, 27.48]
		1998–2002: 31.14 [−9.09, 65.50]
		2002–2022: −1.29 [−9.97, 3.77]
Age ≥ 75 years	1980–1999: −5.14 [−11.45, −2.67]	1980–2000: −9.19 [−12.25, −6.97]
	1999–2022: 2.29 [0.30, 6.80]	2000–2003: 48.69 [8.33, 67.94]
		2003–2022: −1.37 [−5.10, 0.88]
**Mortality**		
All ages	1980–1983: 17.99 [0.10, 50.12]	1980–2022: 1.37 [0.68, 2.06]
	1983–2022: −0.57 [−2.89, −0.12]	
Age < 65 years	1980–2015: 0.59 [−0.35, 2.58]	1980–2022: 1.77 [0.56, 2.99]
	2015–2022: −11.97 [−35.88, −2.69]	
Age 65–74 years	1980–1983: 31.72 [0.03, 102.93]	1980–2022: 1.51 [0.34, 2.72]
	1983–2022: −0.51 [−13.19, 0.55]	
Age ≥ 75 years	1980–2022: −0.13 [−0.80, 0.54]	1980–2022: 1.32 [0.36, 2.27]

^a^ Annual percent change.

^b^ Confidence interval.

The age‐specific analysis showed that up to about 40 years of age, the incidence of liver cancer was very low but increased thereafter and was highest in individuals aged 70 years and older (Figure 2). The analyses stratified by sex and age group (Figure 3) underline these findings with the highest incidence rate observed in the oldest age group (≥ 75 years) in most years.

**FIGURE 2:**
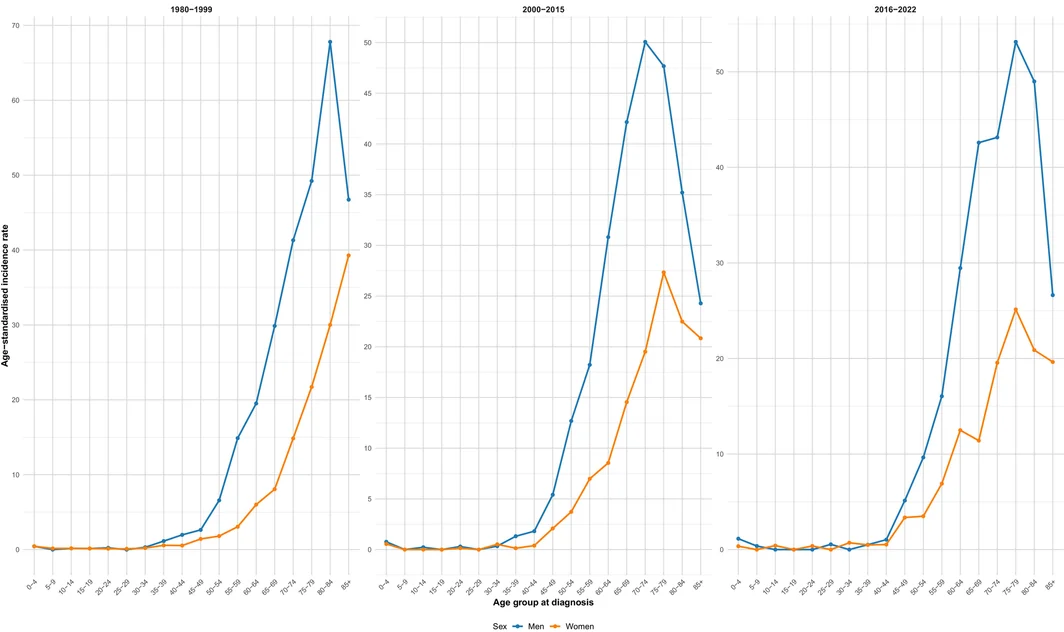
Age‐standardised incidence rates for liver cancer (per 100,000 person‐years) according to five‐year age groups, by sex and period, in the canton of Zurich, Switzerland. The observed values (points) are connected by a line.

**FIGURE 3:**
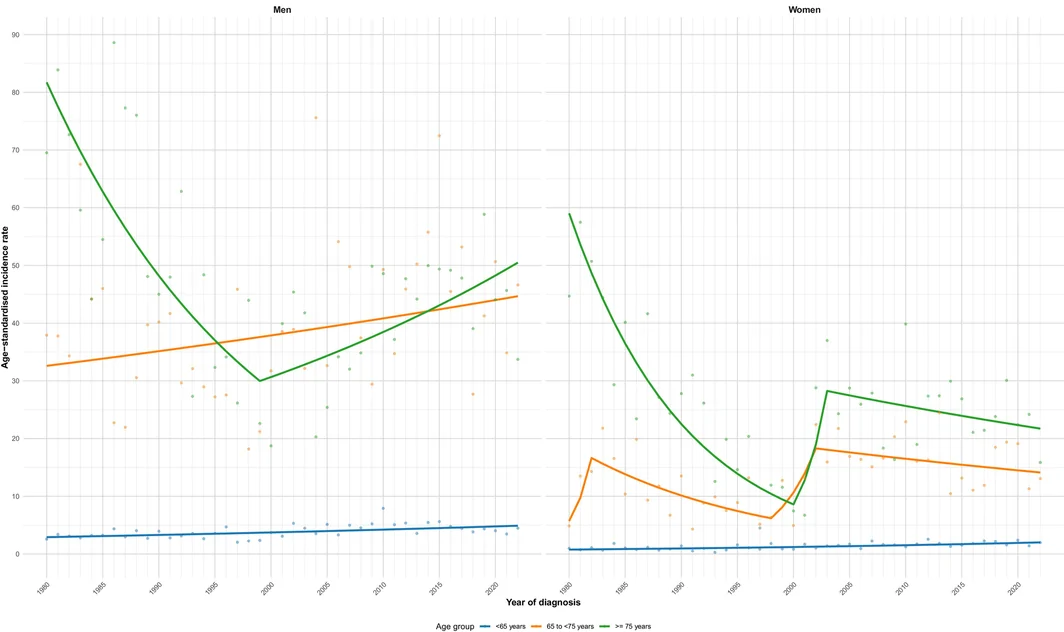
Trends in age‐standardised incidence rates for liver cancer (per 100,000 person‐years) from 1980 to 2022, by sex and age group, in the canton of Zurich, Switzerland. The points show the observed values, and the linear trend line indicates the average percentage change.

Jointpoint regression analysis stratified by age group revealed a significant increase in age‐standardised liver cancer incidence among individuals younger than 65 years throughout the entire study period. The AAPC was 1.2% (95% CI 0.6%–1.9%) for men and 2.3% (95% CI 1.3&–3.3%) for women (Figure 3).

### 3.2 Mortality

Between 1980 and 1999, age‐standardised liver cancer mortality rate was 6.8 per 100,000 person‐years in men and 2.0 in women. In the most recent period (2016–2022), rates were 6.0 and 2.7, respectively. Over the full observation period (1980–2022), the AAPC was 0.7% (95% CI −0.6% to 1.6%) in men and 1.4% (95% CI 0.7%–2.1%) in women, suggesting a stable trend in men and a modest increase in women. Mortality rates consistently remained higher in men than in women throughout the whole study period (Table 3, Figure 4).

**FIGURE 4:**
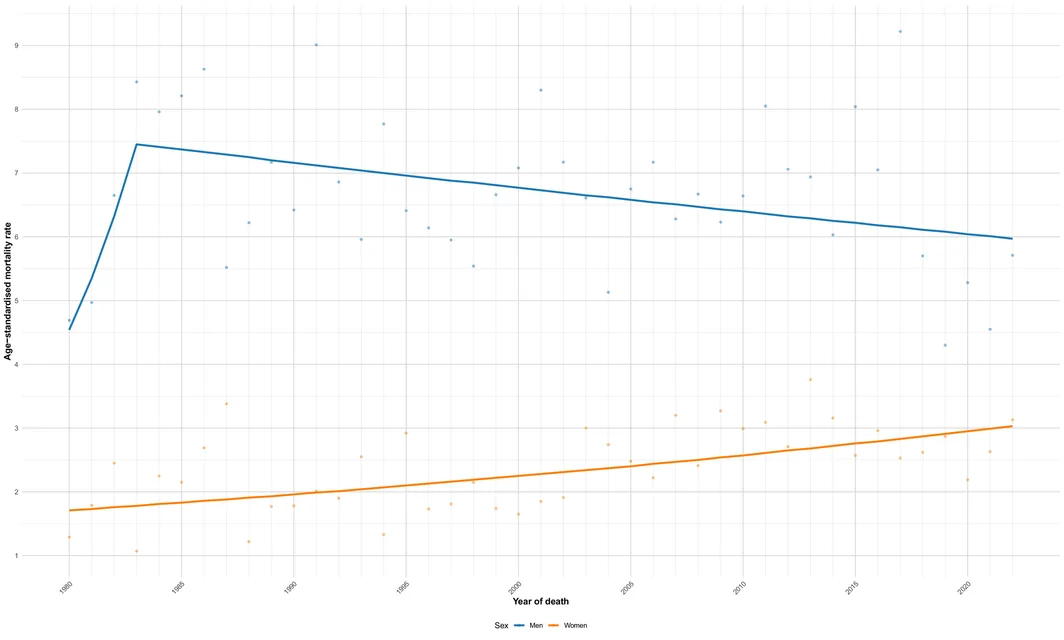
Trends in age‐standardised mortality rates for liver cancer (per 100,000 person‐years) from 1980 to 2022, by sex, in the canton of Zurich, Switzerland. The points show the observed values, and the linear trend line indicates the average percentage change.

Joinpoint regression analysis identified one joinpoint in men, indicating a significant increase until 1983 followed by a subsequent decline, whereas no joinpoint was detected in women, in whom mortality increased significantly and consistently over the study period (Table 4, Figure 4).

In both sexes, liver cancer mortality was very low up to the age of about 40 years, but increased rapidly thereafter (Figure 5). The analyses stratified by sex and age group (Figure 6) underline the findings, with the highest mortality rates observed in the oldest age group (≥ 75 years) and the lowest mortality rates in the youngest age group (< 65 years) throughout the whole study period.

**FIGURE 5:**
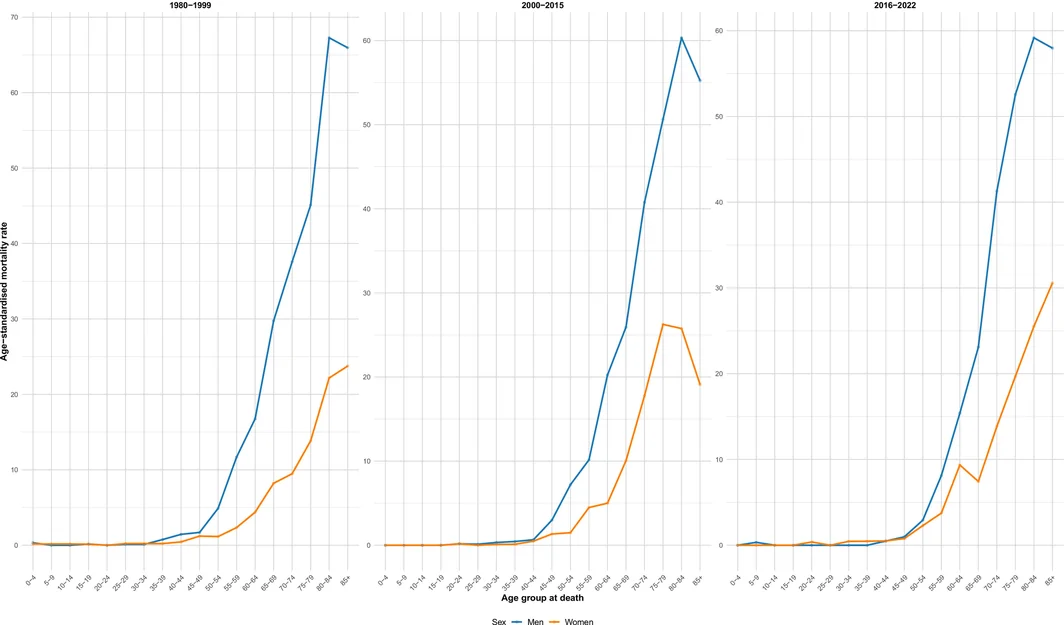
Age‐standardised mortality rates for liver cancer (per 100,000 person‐years) according to five‐year age groups, by sex and period, in the canton of Zurich, Switzerland. The observed values (points) are connected by a line.

**FIGURE 6:**
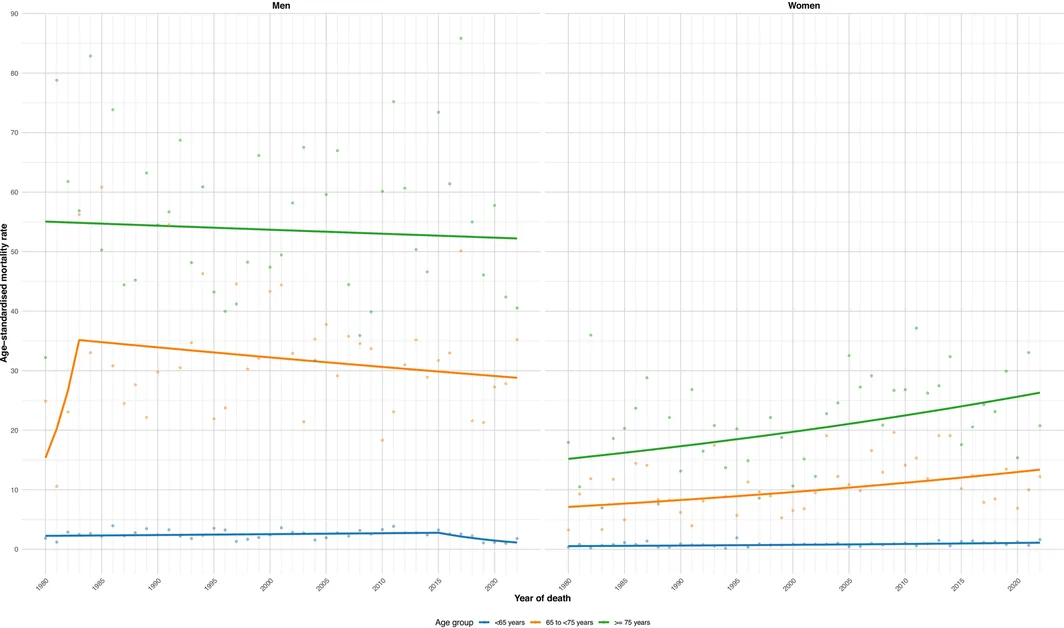
Trends in age‐standardised mortality rates for liver cancer (per 100,000 person‐years) from 1980 to 2022, by sex and age group, in the canton of Zurich, Switzerland. The points show the observed values, and the linear trend line indicates the average percentage change.

### 3.3 One‐ and Five‐Year Relative Survival

Age‐standardised one‐year and five‐year relative survival for liver cancer improved markedly over the study period. Overall, one‐year relative survival increased from 13.8% (95% CI 12.0%–15.9%) in 1980–1999 to 40.0% (95% CI 37.5%–42.5%) in 2000–2015 and 53.9% (95% CI 50.6%–57.4%) in 2016–2022. Five‐year relative survival increased from 3.5% (95% CI 2.5%–4.8%) to 16.2% (95% CI 14.3%–18.5%) and 25.8% (95% CI 22.5%–29.6%) across the same periods (Table 3).

The observed improvement was higher in men than in women. Among men, 1‐year relative survival increased from 13.0% (95% CI 10.9%–15.4%) in 1980–1999 to 56.0% (95% CI 51.9%–60.3%) in 2016–2022, while 5‐year relative survival increased from 2.5% (95% CI 1.6%–3.9%) to 27.9% (95% CI 23.8%–32.8%). Among women, 1‐year relative survival increased from 16.5% (95% CI 13.1%–20.8%) to 49.9% (95% CI 44.4%–56.1%), and 5‐year relative survival from 6.0% (95% CI 3.9%–9.2%) to 21.9% (95% CI 17.0%–28.2%).

## 4 Discussion

This study analysed long‐term epidemiological trends in primary liver cancer cases in the canton of Zurich over more than four decades. Between 1980 and 2022, around 4000 incident liver cancer cases were documented—predominantly hepatocellular carcinoma—alongside almost 3400 deaths due to liver cancer. The proportion of HCCs in our cohort was 71.3%, which is lower than the approximately 90% generally reported for primary liver cancers. This difference is likely attributable, at least in part, to differences in local morphology coding. In particular, the relatively high proportion of cases classified as unspecified (5.5%) and the comparatively high proportion of intrahepatic cholangiocarcinoma (iCCA; 19.3%) may contribute to the observed discrepancy and should therefore not necessarily be interpreted as a contradiction.

Throughout the study period, incidence and mortality rates were consistently higher in men than women. Notably, incidence rates increased among individuals younger than 65 years, while mortality rates rose significantly among women only. One‐ and five‐year relative survival improved markedly over time, reflecting gradual progress in early detection and treatment outcomes.

### 4.1 Comparison With Other Studies

#### 4.1.1 National Perspective (Switzerland)

According to the data from the National Institute for Cancer Epidemiology and Registration (NICER), incidence and mortality rates of primary liver cancer in Switzerland increased only modestly between 1980 and 2010. NICER also reported progressive improvements in short‐term survival. Between 1990 and 1999, the age‐standardised relative one‐ and three‐year survival rates reached 27.9% and 11.8% in men and 29.8% and 11.6% in women, respectively; these improved to 40.2% and 19.9% in men and 42.3% and 19.4% in women between 2000 and 2009. These national figures confirm the gradual improvement in survival outcomes observed in Zurich [24].

#### 4.1.2 Regional Comparison (Europe)

Across Europe, marked variation exists in liver cancer incidence and mortality. In 2020, age‐standardised incidence rates ranged from 4.3 per 100,000 person‐years in Central Eastern Europe to 5.4 in Western and 6.7 in Southern Europe. Western European countries such as France, Spain, and Italy—previously those with the highest incidence—reported lower rates in 2019 compared to 2010, whereas modest increases were observed in Northern Europe. The Global Burden of Disease study attributed these diverging trends to shifts in aetiology, with alcohol‐related liver disease and metabolic dysfunction‐associated steatotic liver disease (MASLD) increasingly contributing to the liver cancer burden, while viral hepatitis‐related liver cancer has declined in several regions following vaccination and antiviral therapies [25].

Italy, historically characterised by high hepatitis C virus (HCV) prevalence, has shown a decline in liver cancer incidence following widespread direct‐acting antivirals use [26], while Nordic and Central European countries may face future increases in liver cancer burden related to the growing prevalence of obesity, metabolic dysfunction, and MASLD [27].

This broader European trend is supported by a population‐based Dutch study investigating hepato‐pancreato‐biliary cancers between 1989 and 2022. The study reported a steady rise in liver cancer incidence—from 3.0 to 8.2 per 100,000 person‐years in men and from 1.2 to 3.9 in women—mirrored by concurrent increases in mortality. Relative 5‐year survival improved markedly from 3%–6% to around 18%–21%, consistent with the positive survival development seen in our Zurich cohort and across Western Europe [28].

In France, a comprehensive analysis of patients diagnosed with liver cancer between 2009 and 2012 revealed a median survival of 9.4 months (95% CI 9.2–9.8 months) post‐diagnosis, with actuarial survival rates of 45.4% at 12 months, 22.8% at 36 months, and 9.6% at 60 months [29].

A follow‐up study analysing trends in survival of primary liver cancer in the United Kingdom between 2000 and 2021 reported survival patterns comparable to those in our study. One‐year survival increased from 35.6% (95% CI 30.5%–41.5%) in 2000–2004 to 42.7% (36.9%–49.4%) in 2020–2021. Among men, 1 year‐survival rose from 33.2% (27.2%–40.4%) to 45.5% (38.8%–53.4%), whereas in women it declined from 40.6% (31.7%–51.9%) to 34.6% (24.3%–49.3%). Similarly, five‐year survival increased from 12.0% (8.7%–16.6%) in 2000–2004 to 15.6% (13.0%–18.8%) in 2015–2019. In men, five‐year survival improved from 9.6% (6.1%–15.0%) to 16.1% (13.1%–19.8%), while in women it decreased from 17.2% (10.8%–27.4%) to 14.6% (9.9%–21.8%) [30].

#### 4.1.3 Global Trends

At the international level, the Global Burden of Disease analyses have consistently demonstrated pronounced geographic variation in primary liver cancer incidence and mortality. Between 1990 and 2016, global age‐standardised incidence increased from 18.4 to 22.3 per 100,000 person‐years in men and from 7.2 to 7.5 in women [31]. The 2020 update estimated a global incidence rate of 9.5 per 100,000 person‐years with the highest burden in Eastern Asia (17.8 per 100,000 person‐years) and the lowest in South‐Central Asia (3.0 per 100,000 person‐years). Corresponding global mortality was 8.7 per 100,000 person‐years, with highest rates in Eastern Asia (16.1 per 100,000 person‐years) and lowest rates in South‐Central Asia (2.8 per 100,000 person‐years) [1].

More recent estimates from 2022 refined this picture reporting 866,136 new liver cancer cases worldwide with men accounting for nearly 70% of diagnoses. The global age‐standardised incidence rate was 8.6 per 100,000 person‐years overall (12.7 in men and 4.8 in women). The global mortality burden remained similarly high, with 758,725 deaths and an age‐standardised mortality rate of 7.4 per 100,000 person‐years (10.9 in men and 4.1 in women). Nearly half of all new cases and deaths occurred in East Asia, emphasising the substantial regional disparities in liver cancer burden. The highest age‐standardised incidence rates were reported in Micronesia (15.1 per 100,000 person‐years), Eastern Asia (14.7), and Northern Africa (14.2), while the highest age‐standardised mortality rates occurred in Northern Africa (13.7), followed by Southeastern (13.0), and Eastern Asia (11.9) [32]. Prognosis remains poor, with the maximum relative five‐year survival estimated at 18% in high‐income countries. These global observations underline that men are much more affected by liver cancer than women and highlight geographical variations based on differences in risk factors [6].

### 4.2 Interpretations

The higher incidence rates of liver cancer observed in men compared to women are not yet fully understood. Both biological and behavioural explanations are plausible. Women may benefit from a protective effect of oestrogen and are less likely to smoke or consume alcohol—two well‐established risk factors for liver cancer [33].

The observed increase in liver cancer mortality among women despite relatively stable overall incidence warrants particular attention. Several factors may potentially contribute to this pattern, including differences in disease stage at diagnosis, tumour biology, underlying liver disease, treatment allocation, or access to and receipt of cancer‐directed therapies. Differences in diagnostic pathways may also play a role, including the possibility of delayed diagnosis or differences in the detection of asymptomatic disease. However, these explanations cannot be evaluated directly in the present study because individual‐level information on disease aetiology, detailed tumour characteristics, treatment allocation, comorbidities, and access to care was not available. The observed sex‐specific mortality pattern should therefore be interpreted as an epidemiological finding rather than evidence for a specific underlying mechanism.

The rising incidence among individuals under 65 years of age might reflect changes in the prevalence of metabolic and behavioural risk factors, including obesity, diabetes, smoking and alcohol consumption, particularly in younger populations [34]. The increasing prevalence of metabolic dysfunction and MASLD may therefore represent one potential contributor to these trends. However, because individual‐level data on metabolic risk factors, alcohol consumption, and liver disease aetiology were not available in our registry dataset, the relative contribution of MASLD, MetALD, alcohol‐related liver disease, and other risk factors to the observed increase cannot be determined. In contrast, the incidence of hepatitis B and C infections has declined substantially in Switzerland due to the successful vaccination and preventive measures, and the availability of new antiviral therapies that effectively prevent the chronic progression of these infections [35, 36]. Because chronic HBV and HCV infections often lead to liver cancer many years after infection, the decrease in HBV and HCV infections may have contributed towards stable rather than rising liver cancer incidence rates in older age groups [35, 36].

An additional consideration under the updated SLD nomenclature is metabolic dysfunction‐associated steatotic liver disease with increased alcohol intake (MetALD). MetALD recognises the substantial overlap between metabolic dysfunction and alcohol exposure, which may be particularly relevant in Western populations where obesity, metabolic risk factors, and alcohol consumption frequently coexist [5]. This overlap may complicate the attribution of liver cancer to a single underlying aetiology and suggest that some cases previously classified as NAFLD or alcohol‐related liver disease may now fall within the MetALD spectrum. Given that our registry data did not contain individual‐level information on metabolic risk factors or alcohol consumption, we were unable to directly assess the contribution of MASLD or MetALD to the observed liver cancer trends.

The improved survival rates observed in Switzerland and other countries may reflect a combination of advances in diagnostic imaging, earlier detection, more individualised treatment strategies, and the availability of novel therapeutic approaches. Progress in systemic therapies, including the introduction of sorafenib after 2007 and atezolizumab‐bevacizumab after 2020, as well as advances in liver transplantation and locoregional treatments, may have contributed to improved outcomes. However, individual‐level treatment data and longitudinal information on treatment eligibility and access were not available for our cohort, and the distribution of treatment modalities across periods could therefore not be assessed. Consequently, the contribution of specific therapeutic advances to the observed survival improvement cannot be determined. In addition, broader use and improved sensitivity of cross‐sectional imaging may have resulted in earlier diagnosis and lead‐time bias or stage‐migration, while changes in case ascertainment and an increasing proportion of incidentally detected or asymptomatic small HCCs may also have contributed to the observed survival trends [8, 37].

### 4.3 Strengths and Limitations

Major strengths of this study include the long observation period and the high quality of cancer registry data [38]. In addition, stratified analyses by sex, age group, and study period enabled the detection of subgroup‐specific trends, while the use of age‐standardised rates allowed for meaningful comparisons across time, between studies, and countries. Nonetheless, several limitations merit consideration. The number of cases per incidence year remains relatively small, limiting the precision of some subgroup estimates. This is particularly relevant for the earliest study period (1980–1999), in which the low annual case counts may have resulted in less precise and less stable 5‐year relative survival estimates. These early survival estimates should therefore be interpreted with caution. Additionally, as the analysis was restricted to data from the canton of Zurich, the generalisability of these findings to other regions may be limited.

Furthermore, the registry data did not include individual‐level information on metabolic risk factors, alcohol consumption, or underlying liver disease aetiology. Consequently, the contribution of MASLD, MetALD, alcohol‐related liver disease, and other specific aetiologies to the observed temporal trends could not be directly quantified.

An important limitation of this study concerns the assessment of disease stage. TNM staging was used because it was the staging system available in the population‐based cancer registry. For HCC, however, the Barcelona Clinic Liver Cancer (BCLC) system [39] is the established clinical framework for prognostic classification and treatment allocation, as it incorporates tumour burden together with liver function and performance status. BCLC staging could not be reliably reconstructed in our cohort because key variables required for its assignment, particularly Child‐Pugh or ALBI grade and performance status, were not routinely recorded in the registry.

Furthermore, TNM stage was missing for 58.8% of cases diagnosed between 2003 and 2022, despite TNM staging being systematically recorded during this period. This substantial proportion of missing staging information represents an important limitation and restricts the interpretations of stage distributions and stage‐specific temporal trends. The reasons for missing TNM stage could not be determined from the registry data; therefore, missingness cannot be assumed to be random, and systematic differences in stage ascertainment across study periods or clinical settings cannot be excluded. Consequently, changes in TNM stage distribution should be interpreted cautiously and should not be considered a complete representation of changes in disease severity over time. In particular, TNM‐based staging should not be interpreted as a substitute for BCLC classification in HCC.

Another limitation concerns the comparability of mortality data over the study period due to the transition from ICD‐8 to ICD‐10 in 1995. Mortality cases were defined using ICD‐8 code up to 1994 and ICD‐10 codes C22.0 and C22.1 from 1995 onward. Although these codes were used consistently with the mortality case definition applied in the study, changes in coding practices associated with the transition between classification systems may affect comparability across the entire observation period and could potentially introduce a discontinuity around 1995. Therefore, long‐term mortality trends should be interpreted with some caution around the coding transition. No additional sensitivity analysis was performed.

Finally, the most recent study period includes the years 2020–2022, which coincided with the COVID‐19 pandemic. The pandemic may have affected healthcare utilisation, diagnostic pathways, cancer detection, treatment delivery, and cancer registration, potentially influencing incidence and survival estimates during this period. Evidence from Swiss cancer registry data supports this concern. Suter et al. reported a decrease in cancer diagnoses during the first year of the pandemic, particularly during the most stringent lockdown phase in April 2020, with some cancer incidence rates increasing again in 2021. In a subsequent analysis, they also observed differences in cancer stage distribution and longer times to treatment initiation for some treatment modalities among patients diagnosed during the pandemic compared with the pre‐pandemic period. These findings suggest that disruptions in cancer detection and treatment may have affected cancer outcomes during the pandemic period. However, the extent to which such effects influenced the estimates in the present cannot be quantified because individual‐level information on pandemic‐related delays in diagnosis or treatment was not available. Moreover, changes observed in Swiss cancer registry data during this period may also partly reflect the implementation of the new Swiss Cancer Registration Act in 2020. Therefore, estimates for 2020–2022 should be interpreted with some caution, particularly when considering short‐term changes at the end of the observation period [40, 41].

## 5 Conclusions

This long‐term analysis revealed a rising incidence of liver cancer among individuals younger than 65 years of age and an increasing mortality rate among women between 1980 and 2022. These trends may reflect changes in several established risk factors, including metabolic dysfunction and MASLD, alcohol consumption, and chronic viral hepatitis infections. However, because detailed information on these risk factors was not available, their respective contributions to the observed trends could not be directly assessed.

Strengthening primary prevention efforts remains essential to counter these trends. Public health initiatives targeting obesity reduction, promotion of moderate alcohol consumption, and the expansion of hepatitis B vaccination coverage could lower future liver cancer incidence. Broader implementation of screening programmes for high‐risk groups—such as individuals with chronic liver disease—would further support earlier diagnosis and improve treatment outcomes.

Encouragingly, 1‐ and 5‐year relative survival for liver cancer have improved steadily over the past decades. This progress may reflect advances in diagnostic imaging, earlier detection, wider availability of liver transplantation, locoregional treatments, and systemic therapies, including molecularly targeted agents and immunotherapy. However, because detailed treatment data were unavailable, the contribution of specific therapeutic advances to the observed survival improvement cannot be determined. Moreover, multidisciplinary management integrating hepatology and oncology expertise appears to have optimised patient care and improved clinical outcomes. Ongoing research should continue to evaluate how evolving therapeutic approaches and preventive strategies can further reduce the burden of liver cancer in Switzerland and beyond. Continued monitoring of national trends will be essential to assess the effectiveness of prevention strategies and therapeutic innovations.

## Author Contributions

**Nina Zuber:** conceptualization, software, methodology, writing – original draft, formal analysis, visualization. **Flurina Suter:** methodology, software, validation, writing – review and editing. **Miriam Wanner:** data curation, writing – review and editing, conceptualization. **Dimitri Korol:** data curation, writing – review and editing, conceptualization. **Sabine Rohrmann:** conceptualization, methodology, project administration, writing – review and editing, supervision, resources.

## Funding

The authors have nothing to report.

## Ethics Statement

In the Canton of Zurich, all cancer cases are registered with presumed consent and registered based on a decision from 1980 by the Zurich Government Council and the general registry approval from 1995 by the Federal Commission of Experts for professional secrecy in medical research. In this analysis, all data were used anonymously, and therefore, no approval was required from the Ethics Committee of the Canton of Zurich.

## Conflicts of Interest

The authors declare no conflicts of interest.

## Data Availability

The data that support the findings of this study are available on request from the corresponding author. The data are not publicly available due to privacy or ethical restrictions.
